# Plain packaging of tobacco products: Lessons for the next round of implementing countries

**DOI:** 10.18332/tid/130378

**Published:** 2020-11-17

**Authors:** Joanna E. Cohen, Suzanne Zhou, Mark Goodchild, Shane Allwright

**Affiliations:** 1 Department of Health, Behavior and Society, Johns Hopkins Bloomberg School of Public Health, Baltimore, United States; 2 McCabe Centre for Law and Cancer, Melbourne, Australia; 3 Health Promotion Department, World Health Organization, Geneva, Switzerland; 4 Centre for Global Health, School of Medicine, Trinity College Dublin, The University of Dublin, Dublin, Ireland

**Keywords:** plain packaging, tobacco products, standardized packaging, tobacco control policy, low and middle income countries

Australia was the first country in the world to implement tobacco plain and standardized packaging, with plain packs appearing on retailer shelves in December 2012. Plain packaging laws standardize the appearance of packs by prohibiting all design features (including colours, shapes, images, logos, textures/finishes, scents, and promotional text) other than those explicitly permitted. A brand and variant name in a plain font may appear on the pack, and the packs are also required to carry health warnings labels plus other required consumer information. Packs must appear in standard colours, with Pantone 448C, a drab green/brown colour, as the main background colour of the pack^1^.

In June 2020, the final remaining legal challenge to Australia’s tobacco plain packaging laws was decided in favour of Australia. The World Trade Organization’s (WTO’s) Appellate Body found that tobacco plain packaging contributed to its objective of reducing tobacco use and exposure, that it was not more trade-restrictive than necessary to achieve that public health objective, and that it did not infringe any intellectual property rights under the WTO Agreements^2^.

The Appellate Body’s decision ends a decade of litigation against Australia’s tobacco plain packaging laws. This litigation included a challenge by tobacco companies in the High Court of Australia (decided in Australia’s favour in 2012)^3^, an investor–state dispute brought by Philip Morris Asia (PMA) under a bilateral investment treaty with Hong Kong (dismissed in 2015 on the grounds that PMA had abused its rights under the treaty)^4^, and a challenge brought in the World Trade Organization, which was decided in Australia’s favour at the first instance in 2018^5^ and has now been confirmed on appeal. Unsuccessful legal challenges were also launched against plain packaging in the UK^6^, France^7^, Norway^8^, and Ireland^9^. These legal challenges formed part of a broader campaign by the tobacco industry to stop or delay the implementation of plain packaging. The challenges have now been definitively ended by the WTO Appellate Body, whose status as the final appeal mechanism for the multilateral trading system should give other countries the confidence to move ahead with the measure without fear of challenges under trade or intellectual property law.

In addition to litigation, the tobacco industry has used other tactics to oppose plain packaging legislation such as: lobbying; PR/media campaigns in both print and online media (using familiar arguments such as the ‘Nanny state’); threatening manufacturing plant closures, claiming increased illicit trade and increased stealing from retailers; and using third-party front groups to make its arguments^10,11^.

When it cannot prevail in stopping the implementation of plain packaging, the tobacco industry ensures it exploits any loopholes, for example by introducing brand names with colour or concept descriptors^12-14^.

Australia conducted rigorous and extensive research to inform the specifics of their plain packaging requirements and most other countries that subsequently introduced plain packaging legislation adopted or adapted similar requirements^15^. However, while there are many similarities, there are also differences in countries’ specific plain pack requirements, such as differences in the products covered (e.g. most countries exclude e-cigarettes), pack dimensions, and pack edges.

In 2019, Thailand was the first low-to-middle income country (LMIC) to introduce plain packaging and its experiences may be helpful to other LMICs. Due to intense lobbying by the tobacco industry, with Philip Morris International and various front groups arguing that plain packaging was a violation of trademark and intellectual property rights, the Thai legislation was delayed for seven years. When the WTO finally found in favour of plain packaging in June 2018, Thailand moved quickly. The Thai plain packaging regulation was drafted over the next few months and came into effect by September 2019.

As of October 2020, 17 countries have adopted plain packaging: Australia, Canada, France, Ireland, Israel, New Zealand, Norway, Saudi Arabia, Singapore, Slovenia, Thailand, Turkey, UK, Uruguay, Belgium, Hungary, and the Netherlands. Many more have progressed plain packaging laws and regulations to varying extents.

## Conclusions

Plain packaging addresses the obligations to implement effective packaging and labelling measures under Article 11 of the WHO Framework Convention on Tobacco Control (FCTC) and to comprehensively ban tobacco advertising, promotion and sponsorship under FCTC Article 13. There is no need for countries to reinvent the wheel; they can follow the example of other countries with regard to the wording of their legislation as well as arguments and strategies to counteract tobacco industry opposition. There are also valuable resources that countries can use, including plain packaging evidence summaries and toolkits such as the Campaign for Tobacco-Free Kids’ Plain Packaging Toolkit^16^, WHO’s report on the evidence, design and implementation of tobacco product plain packaging^17^, and Cancer Council Victoria’s Plain Facts^18^.

While legal challenges against plain packaging have been unsuccessful, great care is nonetheless required in drawing up legislation. Those involved in drafting plain packaging legislation should work with lawyers and others with experience in countries that have already enacted plain packaging to ensure that their plain packaging legislation is strong and that potential loopholes are minimized.

Plain packaging has great potential globally to change societal perceptions and thereby denormalise use of tobacco products especially among youth, who are vulnerable to tobacco industry marketing strategies. This is particularly important in LMICs where the tobacco industry is aggressively marketing their products. Many LMICs therefore have a unique opportunity to prevent further growth of the tobacco epidemic through plain packaging along with other evidence-based tobacco control measures. It is now time for all countries to move forward with tobacco plain packaging to help save lives by reducing tobacco-related death and disease.
